# Human Pluripotent Stem Cells for Modeling of Anticancer Therapy-Induced Cardiotoxicity and Cardioprotective Drug Discovery

**DOI:** 10.3389/fphar.2021.650039

**Published:** 2021-04-19

**Authors:** Wendy Keung, Yiu-Fai Cheung

**Affiliations:** ^1^ Dr. Li Dak-Sum Research Centre, The University of Hong Kong, Pokfulam, Hong Kong; ^2^ Department of Paediatrics and Adolescent Medicine, Li Ka Shing Faculty of Medicine, The University of Hong Kong, Pokfulam, Hong Kong

**Keywords:** anticancer therapy, cardiotoxicity, human induced pluripotent cells, cardiomyocytes, pharmacogenomics

## Abstract

Anticancer chemotherapies have been shown to produce severe side effects, with cardiotoxicity from anthracycline being the most notable. Identifying risk factors for anticancer therapy-induced cardiotoxicity in cancer patients as well as understanding its underlying mechanism is essential to improving clinical outcomes of chemotherapy treatment regimens. Moreover, cardioprotective agents against anticancer therapy-induced cardiotoxicity are scarce. Human induced pluripotent stem cell technology offers an attractive platform for validation of potential single nucleotide polymorphism with increased risk for cardiotoxicity. Successful validation of risk factors and mechanism of cardiotoxicity would aid the development of such platform for novel drug discovery and facilitate the practice of personalized medicine.

## Introduction

Cancer is one of the leading causes of morbidity and mortality worldwide. Advances in diagnosis and management have reduced death rate of cancer patients significantly over the past decade (Jemal et al., 2010; Silverman et al., 2010). However, a significant number of patients experience severe and debilitating side-effects from anticancer therapies. As survival rate in cancer patients continue to increase, the burden of side effects is also on the rise and remains a grave concern.

Cardiovascular toxicities are amongst the most debilitating side effects of anticancer therapies. Both traditional chemotherapies as well as newer targeted chemotherapies appear to cause different degrees of cardiotoxicities via various mechanisms. Traditional anticancer chemotherapies include anthracycline treatment with doxorubicin as the most commonly used agent (Krischer et al., 1997; van Dalen et al., 2006). Although effective (Jemal et al., 2010; Silverman et al., 2010), doxorubicin has been shown to produce severe side effects with cardiotoxicity being the most notable (Oeffinger et al., 2006; Mulrooney et al., 2009; Lipshultz and Adams, 2010; Tukenova et al., 2010). Novel targeted anticancer therapies may offer a lower incidence of cardiotoxicity, which is also often reversible. Cardiotoxicity of anticancer therapies is highly patient-specific. Several factors need to be taken into consideration when choosing anticancer therapy, including the pharmacodynamics and pharmacokinetics of the drug, as well as pharmacogenomics of the patient. In this review, we shall discuss the use of human pluripotent stem cell technology to study the mechanism of anticancer drug-induced cardiotoxicity, and development of a preclinical human cell-based model of personalized medicine for prediction of adverse effects of anticancer therapies and discovery of novel cardioprotective agents.

## Mechanism of Anticancer Drug Induced Cardiotoxicity

**FIGURE 1:**
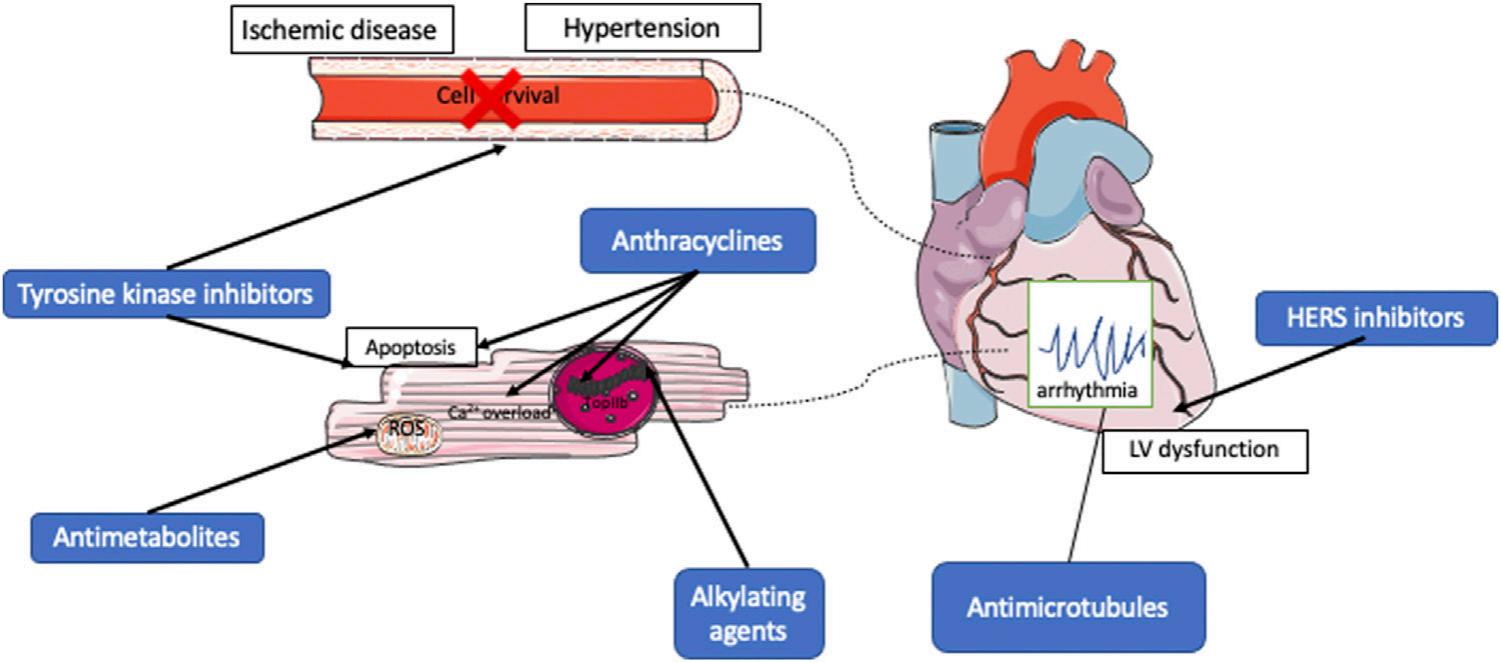
Mechanisms of cardiotoxic effects of anticancer therapies. Anthracyclines may induce cardiotoxicity by increasing ROS production, inhibiting topoisomerase II-ß or by inducing calcium overload in cardiomyocytes. Alkylating agents cause DNA damages in cardiomyocytes. Antimetabolites increase ROS production causing apoptosis in cardiomyocytes while antimicrotubules inhibit cell division and causes arrhythmia. HER2 inhibitors cause myocardial dysfunction by rendering cardiomyocytes incapable of repairing against myocardial stress. Small molecule tyrosine kinase inhibitors cause hypertension and ischemic heart disease by inhibiting nitric oxide production and signaling in blood vessels of the heart and causing mitochondrial damage in cardiomyocytes.

Understanding the fundamental mechanisms underlying anticancer therapy-induced cardiotoxicity is essential to the development of novel approaches to monitor, treat, and prevent these adverse side effects. Both traditional anthracyclines and novel targeted anticancer therapies have been shown to cause varying degrees of cardiotoxicities (Figure 1). However, their mechanisms of pathophysiology are different.

### Anthracyclines

Anthracyclines, including doxorubicin and daunorubicin, have been used to treat a wide range of cancers, including hematological malignancies, solid tumors including neuroblastoma, nephroblastoma, osteosarcoma and Ewing’s sarcoma, in both children and adults with success. Since the introduction of anthracycline to clinical practice in the early 1960s, the overall 5-years survival rates for all childhood cancers have risen from 58 to 83% in 2011 (Miller et al., 2016). Though this class of drug has been in use for over 5 decades, the mechanism of anthracycline-induced cardiotoxicity (AIC) remains understudied. Nonetheless, several hypotheses have been proposed to explain the pathophysiology. One theory is that anthracycline exposure induces the generation of reactive oxygen species (ROS), leading to DNA, protein and lipid damage and subsequent membrane damage and cell death (Deavall et al., 2012). This increase in ROS production, including superoxide and hydrogen peroxide, has been shown to involve iron, which forms an iron-anthracycline complex that catalyzes the conversion of hydrogen peroxide to hydroxyl radical (Minotti et al., 1999; Xu et al., 2005). A second hypothesis involves the inhibition of topoisomerase II-β (TOP2B) and topoisomerase I mitochondrial (TOP1MT) in cardiomyocytes. Different from topoisomerase II-α, which is responsible for the anti-tumor effect of anthracyclines, these two enzymes regulate transcriptional modulation of nuclear and mitochondrial genes and DNA-damage-induced apoptosis (Zhang et al., 2012) by preventing the religation of DNA, leading to the formation of DNA double-strand breaks. TOP2B has been associated with a reduction in mitochondrial biogenesis that is mediated by peroxisome proliferator-activated receptor gamma (PPARG) coactivator 1-α and (PPARGC1A) and PPARG coactivator 1-β (PPARGC1B) (Zhang et al., 2012). In addition, the mitochondrial dysfunction and cell death caused by TOP2B has also been shown to be due to the activation of p53 MAP kinase triggered by double-stranded DNA breaks (Nebigil and Desaubry, 2018). A third hypothesis proposes that doxorubicin and its metabolite doxorubicinol can induce Ca^2+^ release from the sarcoplasmic reticulum (Arai et al., 2000), thereby causing Ca^2+^ overload that leads to sarcomeric disarray and myofibril deterioration (Hanna et al., 2014) and arrhythmia.

### Alkylating Agents

Alkylating agents including cyclophosphamide and cisplatin are amongst the oldest class of anticancer agents. Clinically, they are often used in combination with anthracycline to treat various cancers including lymphoma, multiple myeloma, breast cancer and lung cancer. They act by binding to negatively charged DNA sites, causing DNA strand breakage and cross-linking (Espinosa et al., 2003). These compounds are known to have cardiotoxic effects due to its DNA damaging effect, which can result in apoptosis of not only tumor cells but also normal tissues.

### Antimetabolites and Antimicrotubules

Taxanes, including paclitaxel and docetaxel, are a group of chemotherapeutic agents isolated from natural sources. They are commonly used to treat gynecological and breast cancers as well as lymphoma. They act by binding to microtubules, leading to their polymerization and inactivation, thereby inhibiting cell division. Cardiac arrhythmias and ischemia are the most common cardiotoxic side effects observed in patients receiving paclitaxel. The arrhythmias are related to the effect of paclitaxel on accelerated release of calcium (Zhang et al., 2010). Paclitaxel treatment causes bradycardia in up to 30% of patients and cardiac ischemia in 5% of patients (Rowinsky et al., 1991; Arbuck et al., 1993; Zhang et al., 2010), whereas docetaxel also caused myocardial ischemia albeit at a lower incidence rate of 1.7%. The antimetabolite 5-fluorouracil (5-FU) can induce apoptosis and autophagy through production of oxidative stress in cardiomyocytes and endothelial cells (Freeman and Costanza, 1988; Eskandari et al., 2015).

### Novel Anticancer Therapies

Besides traditional anticancer therapies, novel targeted therapies are in increasing use to treat various types of cancers. Similar to traditional anticancer therapies, patient-specific targeted anticancer therapies also cause on-target or off target cardiotoxicities varying from hypertension, rhythm disturbances, QTc prolongation to ventricular dysfunction. Compared to traditional anticancer therapies, however, these side effects caused by novel anticancer therapies are rarely life-threatening, have no dose-dependent effect and are often reversible upon cessation of treatment (Chen and Ai, 2016).

#### HER2 Inhibition

A portion of breast cancers are HER2-positive. Overexpression of HER2 promotes tumorigenesis (Slamon et al., 1987). In these patients, HER2 inhibition prevents cell repair and significantly limits proliferation of metastatic HER2-positive breast cancers. Trastuzumab is a humanized anti-HER2 monoclonal antibody that targets the extracellular domain of the receptor. It is effective in treating both primary as well as metastastic breast cancers and is the first therapeutic antibody targeting molecular markers in cancer approved by the Food and Drug Administration (FDA). It is used as a first-line therapy either alone or together with other traditional chemotherapies including paclitaxel (Jelovac and Emens, 2013). Trastuzumab blocks the HER2/ErbB2 signaling in cardiomyocytes, rendering them incapable of utilizing the repair mechanism for protection against myocardial stress (Monsuez et al., 2010). In addition, trastuzumab has been shown to cause more pronounced cardiotoxicity when in the presence of anthracyclines (Bartsch et al., 2007; Hahn et al., 2014).

#### Small Molecule Tyrosine Kinase Inhibitor (TKI)

Small molecule TKIs have been developed against a number of targets including vascular endothelial growth factor receptors (VEGFR), platelet-derived growth factor receptors (PDGFR), epidermal growth factor receptor (EGFR) and other kinases. Imatinib is the first FDA-approved TKI for treatment of chronic myelogenous leukemia and gastrointestinal stromal tumors (Druker et al., 2001; Demetri et al., 2002). TKIs that inhibit VEGFRs have been shown to cause various degrees of congestive heart failure. Imatinib, which is a small molecule inhibitor against Abelson family of nonreceptor tyrosine kinases (ABL) with other known targets that include proto-oncogene receptor tyrosine kinase (KIT) and PDGFR, is mainly used to treat chronic myeloid leukemia, has been found to cause congestive heart failure symptoms in 1.7% of patients (Atallah et al., 2007). Imatinib has been demonstrated to cause dilated sarcoplasmic reticulum with membrane whorls, and abnormal mitochondria with effaced cristae by inhibition of ABL tyrosine kinase (Kerkela et al., 2009). Another multiple TKI sunitinib, which targets VEGFR receptors, is overall well-tolerated but may cause adverse cardiovascular effects including congestive heart failure with an incidence ranging from 1.5 to 14% (Richards et al., 2011; Hall et al., 2013) and hypertension with an incidence of 17–43% (Chu et al., 2007). A common mechanism of cardiac dysfunction between VEGFR inhibitors is the activation of hypoxia induced factor-1-alpha (HIF-1α) in response to VEGF induced decrease in capillary density (Ky et al., 2013). The common adverse effect of hypertension is thought to be due to a decreased nitric oxide signaling and increased endothelin-1 production. Sunitinib may have off-target mechanisms, inhibiting AMP-activating protein kinase which normally activates and stimulate ATP production through catabolic pathways (Kerkela et al., 2009). Additionally, PDGFR is also a target of sunitinib and may affect cardiac function in response to mechanical stress. Sunitinib, pazopanib, and vandetanib also prolong the QT interval and therefore increase the risk of Torsades de pointes (TdP).

## Pharmacogenomics and Gene Polymorphism of Anticancer Therapies

An individual patient’s responses to various chemotherapeutic agents are dependent on the pharmacodynamics and pharmacokinetics of the drug. In turn, polymorphism in target proteins of anticancer drugs may influence the pharmacodynamics of the drugs, while polymorphism in enzymes and transporters involved in metabolizing drugs can alter their expression and activity and affect the drug’s pharmacokinetics (Evans and McLeod, 2003). Thus, identifying polymorphism related to targets in anticancer therapies in patient population may help to predict cardiotoxicities in patients. In addition, identifying risk factors for anticancer drug-induced cardiotoxicity in cancer patients as well as understanding its underlying mechanism is essential to novel drug discovery as well as improving clinical outcome of chemotherapy treatment regimens. This allows clinicians to screen patients for susceptibility variants prior to treatment and select an alternative treatment or co-administer protective adjuvant therapy or development modified chemotherapeutics that bypass off-target pathways. Candidate gene association studies (CGAS) in which frequencies of genetic variants, mainly single nucleotide polymorphisms (SNPs) are compared between cases and controls, and genome-wide association studies (GWAS), in which genetic variations across the whole genome are analyzed, are 2 widely used methods to identify variants that may contribute to drug-specific risk factors (Pinheiro et al., 2020). However, while these studies help identify numerous genetic variants, they need further validations in order to determine the causal relationship between genetic variants and occurrence of cardiotoxicities, which is essential for further development of protective therapy (Knowles et al., 2018).

### Anthracycline

Four variant SNPs, CELF4 rs1786814, RARG rs2229774, SLC28A3 rs7853758 and UGT1A6 rs17863783 currently have the strongest and the most consistent evidence for association with AIC (Aminkeng et al., 2016; Wang et al., 2016; Scott et al., 2018). RARG, which codes for retinoic acid receptor gamma, normally acts to repress the expression of TOP2B in the heart, which anthracycline binds to cause double stranded DNA breaks. The rs2229774 variant could lead to reduced repression of TOP2B and thus increased anthracycline toxicity (Aminkeng et al., 2015). CELF4 is an RNA binding protein involved in tissue specific, developmentally regulated pre-mRNA splicing. Splice variants of cardiac troponin T bearing an alternative exon 5 are predominately expressed in the embryonic heart and significantly downregulated during development into the adult heart. CELF4 is known to mediate the alternative splicing of the gene TNNT2. CELF4 variants with reduced activity to target TNNT2 pre-mRNA enable the continued expression of embryonic cardiac troponin T in the adult heart. This results in a dual capacity for thin myofilaments to handle increasing calcium concentrations and potentially compromises their contractility and ultimately left ventricular ejection fraction (Tripaydonis et al., 2019). SLC28A3 belongs to a class of solute transporter proteins that have been shown to transport anthracyclines in cancer cells. It is also expressed in the myocardium. The presence of the SLC28A3 RS7853758 is thought to be protective as it reduces the exposure of cardiomyocytes to anthracyclines (Scott et al., 2018). Rs17863783 is a tag marker of the UGT1A6*4 haplotype, which has been reported to cause a 30–100% reduction in enzyme activity (Krishnaswamy et al., 2005a; Krishnaswamy et al., 2005b; Visscher et al., 2013). Reduced UGT1A6-mediated glucuronidation of anthracycline metabolites may lead to accumulation of toxic metabolites resulting in the observed increased risk of AIC. However, UGT1A6 is not expressed in the heart, which suggests that their contribution to cardiotoxicity is not related to direct effects in cardiac tissue.

Other variants including CYP3A5 rs4646450, ABCC2 rs3740066, NQO1 rs1043470, and SLC22A6 rs6591722 variants have also been reported to be associated with anthracycline-induced cardiotoxicity in children (Sagi et al., 2018). These association studies, however, were unable to establish causality of gene-disease relationship. Although several studies have evaluated the influence of patient genetics on anthracycline-induced cardiomyopathy, no SNPs have been validated by functional studies to be clinically useful predictors of cardiotoxicity risk. Reported results about genetic variants’ associations with AIC are conflicting and often could not be replicated in all studies.

### Alkylating Agents

Studies on the influence of genetic variations on the toxicity of alkylating agents are limited. However, polymorphisms in genes related to metabolism and transport of alkylating agents exist. CYP2B6 and CYP2C19 have been shown to influence cyclophosphamide pharmacokinetics in adult patients (Helsby et al., 2010). Patients carrying CYP2B6*6 have been shown to significantly lower cyclophosphamide clearance. However, whether this polymorphism affects the degree of cardiotoxicity remains to be determined (Veal et al., 2016).

### Antimetabolites and Antimicrotubules

Functional polymorphisms of the ABCB1 gene of the ABC membrane transporter, which is involved in the transport of taxanes have been shown to significantly increase toxicity. However, knowledge on the functional significance of these genetic variants of the ABC transporter is still lacking and further investigation is warranted in order to evaluate the significance of these polymorphism in taxane-induced cardiotoxicity (Jabir et al., 2012).

### Novel Anticancer Therapies

Despite the fact that TKI treatment has been proven to cause cardiotoxicity, few candidate biomarkers or risk SNPs for cardiotoxicity have been identified. Nonetheless, a few candidate biomarkers have been suggested based on CGAS. For example, in one study, patients with heterozygous and homozygous variant in the VEGF gene (rs699947, rs833061, rs1570360, rs2101963, rs3025039) had a 1.56 times higher risk of developing bevacizumab-induced hypertension (Leong et al., 2019). In HER2-positive breast cancer patients, there is a significant association for heterozygotes of rs1136201 with increased risk of developing cardiotoxicity (Beauclair et al., 2007). In another study of HER2-positive breast cancers patients, an association for heterozygous and homozygous variant genotypes of rs1136201 has also been found (Roca et al., 2013), although data pooled from multiple studies failed to show increased risk of developing heart failure (Leong et al., 2019). By contrast, in the same study, pooled analysis showed that the presence of rs1058808 SNP was potentially cardioprotective, and reduced the risk of developing heart failure by 31% (Arbuck et al., 1993).

## Human Induced Pluripotent Stem Cells as Model for Novel Drug Discovery

Whereas animal models may fail to reflect the fundamental biology and therefore the cardiotoxic responses of cancer therapies in human, preclinical validation studies aiming at investigating the mechanism of cardiotoxicity of anticancer therapies traditionally has to rely on methodologies using myocardial biopsies. These novel human-tissue based assay models such as human ventricular slices have proven to be superior over animal models, as being of human origin and composed of a heterogenous mixture of cell types means a more sophisticated model without the disadvantage of species difference which can faithfully recapitulate human cardiac physiology at organ level. However, assays using myocardial tissues are both invasive and has a limited source. As such, current preclinical screening assays to detect cardiotoxicity remain suboptimal, without the capability of testing repeated and chronic dosing regimens on patient-derived cardiomyocytes.

Human induced pluripotent stem cell (hiPSCs) technology, on the other hand, has made it possible to experimentally validate the SNPs that have been identified in CGAS and GWAS studies (Knowles et al., 2018). The hiPSCs is a valuable tool for testing hypotheses of mechanisms of anthracycline-induced cardiotoxicity involving multiple cell types as they can be specified into more than one cell or tissue type. Moreover, with the advent of genome engineering such as the clustered regularly interspaced short palindromic repeat (CRISPR) technology, researchers have been able to correct these variants in a dish to further validate the importance of these genetic variants in disease progression. When validated, the data is useful for identifying novel markers and pathways for drug discovery for cardioprotection. Human iPSC-based drug testing models can also provide a reliable platform for identification of novel drugs that are free of cardiac side effects (Magdy et al., 2016). In addition, drug screening in hiPSC-CMs is now required in the Comprehensive *In Vitro* Proarrhythmia Assay (CiPA) guidelines for cardiac drug development owing to a large number of arrhythmic events identified in approved drugs in the post marketing phase (Gintant et al., 2017). The faithful recapitulation of the disease/SNP phenotype also makes hiPSC a tool for personalized medicine to avoid cardiotoxicity. While the application of hiPSC-CMs for personalized predictions of cardiotoxicity is in its infancy, the strength of their use and the promise shown in preclinical cancer drug cardiotoxicity testing has been recognized and recently been discussed in a scientific statement from the American Heart Association (Gintant et al., 2019).

### hiPSC Based Models for Studying Cardiotoxicity

The earliest hiPSC based drug testing models involve cells cultured in 2D. While patient-specific hiPSC-derived cardiomyocytes have been shown to faithfully model clinical phenotypes of genetic cardiac diseases (Liang et al., 2013), culturing these hiPSC-derived cells in 2D affords limited control in tissue architecture, often resulting in sub-physiological or immature properties, including poor electrophysiology and calcium-handling, leading to poor contractile functions, and a predominantly glycolytic metabolic signature. Some of these immature properties have been proven to be difficult to overcome, such as the development of t-tubules and abolition of immature spontaneous membrane potential oscillation (Lundy et al., 2013; Keung et al., 2014; Karakikes et al., 2015). Despite the existence of these immature traits, we and others have utilized hiPSC/hESC derived cardiomyocytes to study the effects of doxorubicin and were able to demonstrate its cardiotoxic effects *in vitro* (Sirenko et al., 2013; Holmgren et al., 2015; Burridge et al., 2016; Cheung et al., 2020) as well as assess and predict cardiotoxicity in patients (Cheung et al., 2020) by measuring a number of parameters that can directly or indirectly assess the functionality and viability of hiPSC-CMs, including cell viability, contractility, single cell electrophysiology and calcium transients, as well as the detection of biomarkers such as cardiac troponins, micro-RNAs (Cheung et al., 2020; Mehrotra et al., 2020), creatine kinase (Shin et al., 2016) etc, often in high content fashion and longitudinal measurements. Burridge et al. demonstrated that patient-specific hiPSC-CMs can recapitulate predilection of breast cancer patients to AIC at the single cell level (Burridge et al., 2016). This study, however, focused only on patient outcome as the phenotype rather than on the validation of specific SNPs. Their findings nonetheless provide promising data on the application of the hiPCS-CM platform for the validation of genetics variants identified in previous candidate gene and genome-wide association studies and discovery of novel cardioprotective agents. In addition to AIC, 2D cultured hiPSC-CMs have also been shown to successfully demonstrate the cardiotoxic effects of TKIs in a high throughput manner (Sharma et al., 2017).

Three-dimensional tissues of hiPSC-CMs have gained increasing use in modeling drug-induced cardiotoxicity. These tissues offer better control and mimicry of the *in vivo* physiological environment for maturation of cells (Li et al., 2018). Two types of 3D tissues have been used as a model to study anticancer therapies. The simpler form is cardiac organoid, which involves self-aggregation of hiPSC-CMs and or other cell types into spherical organoids in 3D with cellular organization comparable to native heart tissue and which can assess parameters such as cell viability and cytotoxicity (Polonchuk et al., 2017). A more sophisticated type of 3D tissue is engineered cardiac tissue which involves tight control of cellular organization with specific scaffold materials and cell density (Mannhardt et al., 2017a; Keung et al., 2019). The improved alignment of these engineered tissue constructs yields more mature cells and more physiologically relevant functional assessment capabilities that can be adapted to high throughput and high content assays, including true twitch forces, pressure-volume relationships, stroke work and macroelectrophysiological parameters (Mannhardt et al., 2017b; Li et al., 2018; Keung et al., 2019). For example, in a recent study using a form of 3D microtissue to model cardiotoxicity, the authors were able to demonstrate an increase in afterload on cardiomyocytes with increased sunitinib-induced cardiotoxicity, highlighting the superiority of 3D models to assess more complex physiological parameters which are not feasible with 2D culture models (Truitt et al., 2018).

Given the mechanisms of cardiotoxicity induced by different anticancer chemotherapies involve cardiomyocytes, blood vessels, in particular endothelial cells, as well as hepatocytes, an ideal model for validating risk factors and drug discovery should include all relevant cell types (McAleer et al., 2019). Microfluidic systems have been used to connect different cell types derived from hiPSC cultured in 2D to allow for interaction between the different cell types. Such organ-on-a-chip system was able to demonstrate a drug’s effect on multiple cell types including hiPSC-CMs and endothelial cells, hepatocytes and neurons, mimicking the effects of the circulation system, metabolism and/or detoxification of drugs as well as sympathetic control respectively (Oleaga et al., 2016; Weng et al., 2020). Other more sophisticated multiple organ-on-chip devices with organ-level functionality have also been designed, where each ‘organ’ is connected to each other with vascularized, endothelium-lined channels, mimicking blood circulation and recapitulating tissue-tissue interfaces, thus modeling organ crosstalk and metabolism of compounds in a single system. Such multi-cell type assay system could also facilitate drug screening by fulfilling the requirement for *in vitro* physicochemical as well as absorption, distribution, metabolism and excretion (ADME) measurements and help predict pharmacokinetic parameters of drugs, which were previously difficult to measure or model *in vitro*, by introducing cell types including liver and kidney, which can mimic processes including metabolism and excretion respectively (McAleer et al., 2019). An assay system combining multiple cell types and 3D tissue engineering technology would further enhance the organ functionality and interaction. However, such sophisticated system may compromise the throughput of the assay. In the future, a tiered assay system that can accommodate both high throughput and high functionality may be employed in order to more accurately test the increasing amount of new anticancer therapies for cardiotoxic effects (Keung et al., 2019).

### Toward Personalized Medicine: Patient-Specific hiPSC Based Model for Drug Discovery and Testing

Various hiPSC based models have been used for testing of the safety and efficacy of anticancer drugs in a quantitative manner. In one study, a high throughput screening of TKIs has been performed using a 2D hiPSC based model, where different aspects of cell toxicity including cell viability, contractility, calcium handling and electrophysiology were measured to determine a ‘cardiac safety index’ for each TKIs used (Sharma et al., 2017). In addition, the effect of insulin and IGF-1 as protective agents against TKI induced toxicity has also been evaluated. The use of a quantifiable parameter to assess drug toxicity may further facilitate decision making by clinician when evaluating the cardiotoxicity risk of anticancer therapies for patients. Interestingly, only hiPSCs from healthy subjects have been used in this study, the effect of different TKIs on hiPSCs from different genetic background remains to be determined.

**FIGURE 2:**
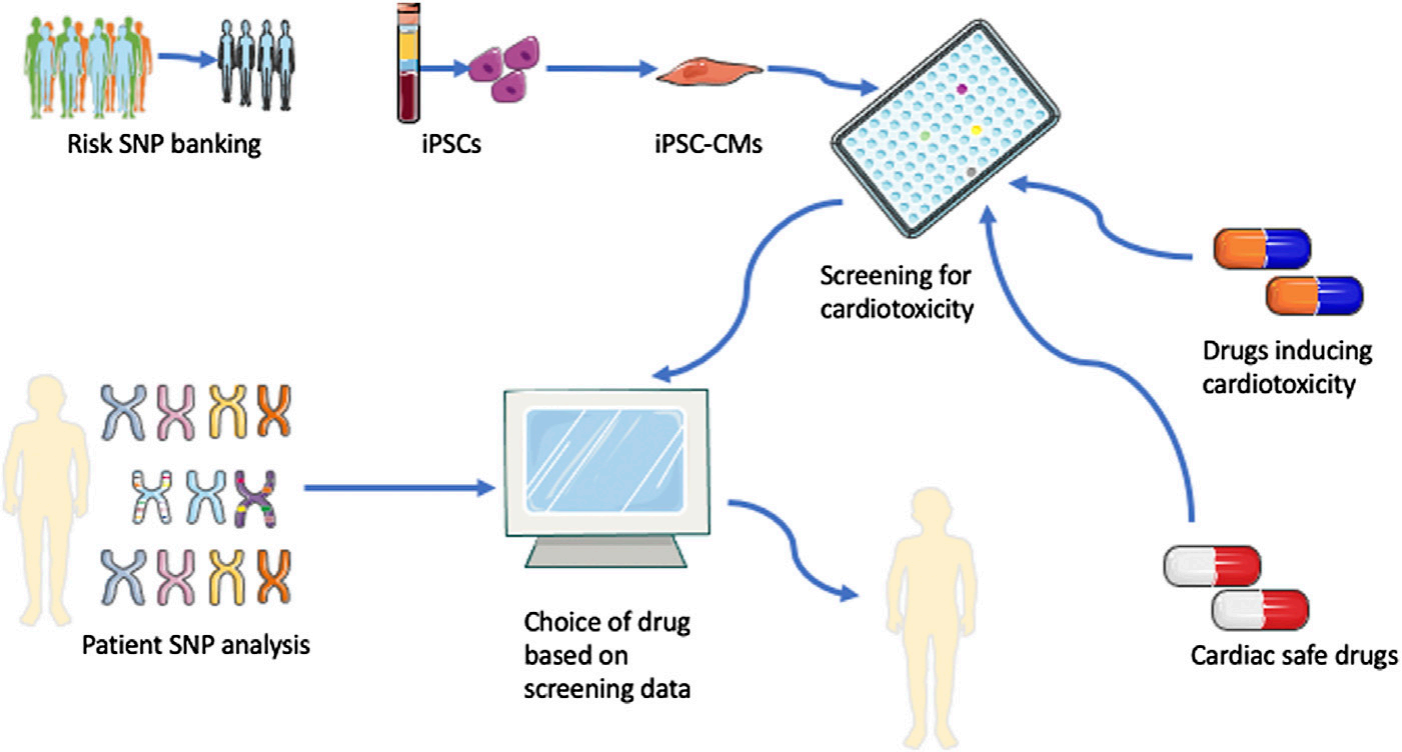
Personalized medicine for anticancer therapy-induced cardiotoxicity. SNP that are high risks for anticancer therapy-induced cardiotoxicity are identified and a biobank of hiPSCs is established. hiPSC from individuals with these high risk SNP can help establish a risk score against each chemotherapeutic agent by screening of these drugs using the hiPSC model. Once assessed, each drug can be assigned a ‘cardiac safety index/score’ unique to each risk SNP. The results can help clinician make informed decision on the choice of anticancer drug with minimal side-effects for better outcome for the patient.

It is conceivable that the hiPSC model could allow a personalized assessment by screening a patient’s cardiomyocytes with different chemotherapeutic agents for prediction of cardiotoxicity. Once assessed, each drug can be assigned a ‘cardiac safety index/score’ unique to the patient in order for the oncologist to make informed decision about the choice of chemotherapy. However, as aforementioned, there are limitations that need to be overcome to enhance the practicality of this approach. One limitation is the time required for the establishment of patient specific hiPSC and their adaptation for drug screening assays. At the current state, this time-consuming process precludes the use of hiPSC for personalized pre-screening of drugs before the choice of chemotherapy has to be made. Alternatively, identifying and validating high risk SNP and establishing biobank of hiPSCs from individuals with these high risk SNP can help establish a risk score for each individual against each chemotherapeutic agent (Sayed et al., 2019), and help clinician to make informed decision on the choice of anticancer drug with minimal side-effects for better outcome for the patient (Figure 2).

## Conclusion

Cardiotoxicity remains to be an important side effect of anticancer chemotherapies. The hiPSC platform offers promise to advance personalized medicine for chemotherapy. With further improvement in maturation of cardiomyocytes and reproducibility of the hiPSC platform, functional validation of pharmacogenomic studies where polymorphisms in genes associated with anticancer drug-induced cardiotoxicity can be achieved. This may provide an invaluable tool to individualize patient-specific chemotherapies and to maximize their benefits and minimal side-effects, thus improving treatment outcome based on the practice of personalized medicine.
